# Convulsions; and the Remarkable Effects of Chloroform

**Published:** 1851-02

**Authors:** Theodore S. Bell

**Affiliations:** Louisville, Ky.


Art. V.—*Convulsions; and the remarkable effects of Chloroform in the Case.* By Theodore S. Bell, M. D., of Louisville, Ky.

Dr. William R. Jacob, of this city, was attacked about the beginning of January, with epileptic convulsions. Several days after the attack, I was requested to visit the case with Dr. Flint. A very remarkable feature presented itself; in the intervals between the spasms; the patient was as motionless and as unconscious as a log. He could neither hear, feel, nor speak, without the use of chloroform, and the inhalation of that always restored him to perfect consciousness, and enabled him to converse with freedom. As soon as the effects of the chloroform passed off, the patient relapsed into utter unconsciousness, from which he could be revived only by chloroform. But I was soon convinced that the use of chloroform was doing more harm than good; the condition of the brain was growing worse under its use, and peremptory directions were given for its discontinuance. In consultation with Dr. Flint, the application of leeches was ordered; they acted well, and I went to the patient, at night, for the purpose of bleeding him, if the leeching had failed to give relief. A brief examination satisfied me that bleeding from the arm was not only indicated, but that it was the only measure that could save the life of the patient. Just as I was preparing to order the bowl and bandages, Dr. J. W. Knight came in, examined the case, and obtained a history of it from me. In our retirement, I stated to him that I was getting ready when he arrived, to bleed from the arm, and he gave his cordial assent to the measure. As soon as the means could be prepared, I bled the patient from a large orifice in the arm, and drew off a large quantity of very black blood, its condition being mainly due to the chloroform.

This bleeding gave great relief to the head, and brought on a crisis in the case. When I reached the patient next morning I was informed he was dying, and there were certainly strong indications of approaching death. The breathing was short and rapid, and frequently suspirous, the pulse almost imperceptible, the eyes glazed, and all the vital powers sinking. By the seasonable use of sinapisms to the arms and legs, hot applications to the feet, and internal stimulants, the untoward symptoms disappeared, and the amendment gave some hope of ultimate recovery. But the subsidense of the convulsive movements left in its train an irritable stomach which rejected every thing that was taken for several days. This irritability of the stomach resisted all attempts at relief, until we gave quinine and creosote, a prescription agreed upon in a consultation between Dr. Flint and myself. The second dose relieved the sickness, and the patient improved rapidly under the use of food. A hemiplegia of the left side, of some months standing disappeared, and the patient emerged from this attack in better condition than he had been in for a considerable length of time.

In dismissing Dr. Jacob from further medical treatment, he was warned to watch for the least aberration from health, and to have it attended to at once. This warning was neglected. On the evening of the 22nd of January, he complained of headache, and gave way to the illusion that it was caused by the noise of some children about the house. On the morning of the 23d he had no appetite, and refused his breakfast, and about 11 o'clock, that day, the spasms returned with great violence. I was immediately summoned to the case, and again resorted to blood-letting from the arm. This attack proved to be much milder than the former one, and was speedily relieved. Although this attack was more the result of spinal irritation, than the former was, the same degree of unconsciousness presented itself, and the chloroform had the same singular effect as that described in the account of the other attack. But in the recent case, the chloroform was used but two or three times, and only for the purpose of putting the physicians in connection, as the Mesmerists say, with the patient. When he was not under the influence of that agent, he seemed perfectly dead to all surrounding objects. When I administered it first in the recent attack, the patient was astonished to hear that he had again been seized with convulsions.

The subject of these attacks of spasms is a young physician, who had generally enjoyed good health before his seizure. On the 15th of last March he was suddenly attacked, when in apparent health; the convulsions were severe, and terminated in hemiplegia. Occasional returns of the convulsions manifested themselves, but the first attack in January was the most formidable the patient had experienced. For a length of time the prognosis was unfavorable. The symptoms of *ramollissement du cerveau* seemed unmistakably present at times. The permanent flexion of the arm of the paralysed side, a tendency to flexion of the leg, and cephalagia fixed to one point, led to the suspicion of softening of the brain, and the absence of other symptoms inclined towards a confirmation of this view. The symptoms during the attack, early in January, very closely resemble those described in the 11th case of Andral's Medical Clinic, on the diseases of the encephalic membranes. But it is evident that the case of Dr. Jacob does not belong to the form of disease described by Andral, for the paralysis disappeared completely, with the disappearance of the other symptoms. What particular agent removed or corrected the lesion in the ventricle or ventricles that caused the hemiplegia, it is difficult to determine. The effect, however, was transient, for the paralysis returned with the recent attack, and remains at this time. It is certainly important that correct conclusions shall be drawn on this point, but the difficulties attendant upon a diagnosis may be estimated from what Andral says, on the lesions, which, in cases of meningitis, coincide with the different alterations in the power of motion. Andral remarks: "beyond that lesion which the scalpel points out as having its seat in the membranes of the brain, there is, in the brain itself, a modification, not recognisable by the anatomist, which is produced, to be sure, by the lesion of the membrane, but which, variable in each individual, is the real cause of all the functional disturbances which are seen to supervene." And it must be remembered that the phenomena described in the case under consideration may be the result of alterations in the substance of the brain itself.

There is reason to hope, whatever the lesion may be, or wherever located, that it is of a removable character. Its sudden and perfect removal in the train of the formidable symptoms that assailed the first attack described in this report, justifies the belief that medication and proper care may again remove it. The patient is now restricted to a nutritious diet, devoid of all stimulants, and the medication relied on is that produced by the Iodide of Potassium. A slight ptyalism is now present, from the use of one dose of calomel. The salivation may aid the Iodide. A further report of the case will be given hereafter, and I entertain a confident hope that the future report will record the recovery of the patient.

January 28th, 1851.

